# Low-touch, team-based care for co-morbidity management in cancer patients: the ONE TEAM randomized controlled trial

**DOI:** 10.1186/s12875-021-01569-8

**Published:** 2021-11-18

**Authors:** Leah L. Zullig, Mohammad Shahsahebi, Benjamin Neely, Terry Hyslop, Renee A. V. Avecilla, Brittany M. Griffin, Kacey Clayton-Stiglbauer, Theresa Coles, Lynda Owen, Bryce B. Reeve, Kevin Shah, Rebecca A. Shelby, Linda Sutton, Michaela A. Dinan, S. Yousuf Zafar, Nishant P. Shah, Susan Dent, Kevin C. Oeffinger

**Affiliations:** 1 Department of Population Health Sciences, Duke University School Of Medicine, 215 Morris St, Durham, NC 27701 USA; 2 Department of Population Health Sciences, Duke University School of Medicine, 411 West Chapel Hill Street, Suite 600, Durham, NC 27701 USA; 3 Duke University Family Medicine and Community Health, 2424 Erwin Rd, Ste 601, DUMC, Box 2714, Durham, NC 27705 USA; 4 Center for Onco-Primary Care, Duke Cancer Institute, 2424 Erwin Road, Hock Plaza, Ste 601, Durham, NC 27705 USA; 5 Duke Cancer Institute, Duke University, 2424 Erwin Rd, Durham, NC 27701 USA; 6 Department of Biostatistics, Duke University, 2424 Erwin Road, 9064 Hock Plaza, Durham, NC 27705 USA; 7 Department of Population Health Sciences, Duke University School Of Medicine, 215 Morris St, Durham, NC 27701 USA; 8 Duke Cancer Network, 20 Duke Medicine Circle, Durham, NC 27710 USA; 9 Duke Institute for Health Innovation, Duke University Health System, 200 Morris St, Durham, NC 27701 USA; 10 Duke Psychiatry and Behavioral Sciences, Duke University School of Medicine, 2200 W. Main St, Ste 340, Durham, NC 27705 USA; 11 Department of Chronic Disease Epidemiology, Yale School of Public Health, 60 College Street, New Haven, CT 06510 USA; 12 Duke University School of Medicine, 2200 W. Main St, Ste 340, Durham, NC 27705 USA; 13 Duke Heart Center, Duke University School of Medicine, 2200 W. Main St, Ste 340, Durham, NC 27705 USA; 14 Duke Cancer Institute, Duke University, 2200 W. Main St, Ste 340, Durham, NC 27705 USA; 15 Duke Cancer Institute, Duke University School of Medicine, 2200 W. Main St, Ste 340, Durham, NC 27705 USA

**Keywords:** Cancer survivorship, onco-primary care, primary care, oncology, health services research

## Abstract

**Background:**

As treatments for cancer have improved, more people are surviving cancer. However, compared to people without a history of cancer, cancer survivors are more likely to die of cardiovascular disease (CVD). Increased risk for CVD-related mortality among cancer survivors is partially due to lack of medication adherence and problems that exist in care coordination between cancer specialists, primary care physicians, and cardiologists.

**Methods/Design:**

The Onco-primary care networking to support TEAM-based care (ONE TEAM) study is an 18-month cluster-randomized controlled trial with clustering at the primary care clinic level. ONE TEAM compares the provision of the iGuide intervention to patients and primary care providers versus an education-only control. For phase 1, at the patient level, the intervention includes video vignettes and a live webinar; provider-level interventions include electronic health records-based communication and case-based webinars. Participants will be enrolled from across North Carolina one of their first visits with a cancer specialist (e.g., surgeon, radiation or medical oncologist). We use a sequential multiple assignment randomized trial (SMART) design.

Outcomes (measured at the patient level) will include Healthcare Effectiveness Data and Information Set (HEDIS) quality measures of management of three CVD comorbidities using laboratory testing (glycated hemoglobin [A1c], lipid profile) and blood pressure measurements; (2) medication adherence assessed pharmacy refill data using Proportion of Days Covered (PDC); and (3) patient-provider communication (Patient-Centered Communication in Cancer Care, PCC-Ca-36).

Primary care clinics in the intervention arm will be considered non-responders if 90% or more of their participating patients do not meet the modified HEDIS quality metrics at the 6-month measurement, assessed once the first enrollee from each practice reaches the 12-month mark. Non-responders will be re-randomized to either continue to receive the iGuide 1 intervention, or to receive the iGuide 2 intervention, which includes tailored videos for participants and specialist consults with primary care providers.

**Discussion:**

As the population of cancer survivors grows, ONE TEAM will contribute to closing the CVD outcomes gap among cancer survivors by optimizing and integrating cancer care and primary care teams. ONE TEAM is designed so that it will be possible for others to emulate and implement at scale.

**Trial registration:**

This study (NCT04258813) was registered in clinicaltrals.gov on February 6, 2020.

## Background

By 2030, there will be over 22 million cancer survivors [1]. Approximately 70% of cancer survivors have cardiovascular disease (CVD) risk factors (e.g., comorbidities such as hypertension and diabetes) that require comprehensive care [2, 3]. Many have a higher risk of mortality from CVD than from cancer and these are often under recognized and under treated [2, 3]. Therefore, effective management of CVD risk is essential for reducing mortality among a growing population of cancer survivors. Due to the intensity of tests and treatments during diagnosis and survivorship, existing models of care generally do not integrate primary care or cardiology in patients with established CVD throughout patients’ cancer treatment continuum. Between 50% to 90% of PCPs care for long-term cancer survivors [4–7], yet there are numerous problems with the existing relationship with cancer specialists, including: suboptimal communication; uncertainty regarding each other’s roles, knowledge, and experiences; and appropriate referrals to other specialists [4, 6–13]. There is often disengagement by primary care providers during the active phase of cancer therapy, and they may or may not be reengaged after therapy is complete.

This lack of coordination of care can be harmful for chronic disease management among cancer survivors. For example, due to advances in screening, early detection, and cancer therapy, the 5-year survival rate in women with breast cancer now exceeds 90% [14, 15], with the survival rate for localized disease nearly 99% [16]. Unfortunately, the increased risk of CVD mortality manifests approximately 7 years after cancer diagnosis [17]. Research has focused on the risk of heart failure due to cancer therapy (e.g., anthracyclines, trastuzumab) [18–22], or coronary artery disease due to left-sided breast irradiation [23, 24]; however most CVD is largely due to aging, obesity, poor lifestyle habits, and other comorbidities like diabetes, hyperlipidemia, and hypertension [25–27]. With a median age of 63 years at time of breast cancer diagnosis, the majority of women will have at least one CVD risk factor [2, 21, 27, 28].

Adding another layer of complexity to the challenge of managing CVD risk is patients’ adherence to CVD-related medications. Among women with early stage breast cancer who were prescribed a statin prior to their breast cancer diagnosis, adherence significantly decreased from 1-year pre-diagnosis (67%) to 2-years post-diagnosis (35% ) [29]. Even by 3-years post diagnosis, the adherence rate (50%) was still substantially lower than the pre-cancer rate. This decrease is also seen with antihypertensive and oral diabetes medications [30]. Not surprisingly, women who are non-adherent with their CVD medications are also more likely to be non-adherent with their post-breast cancer hormonal therapy (e.g. aromatase inhibitor ) [31]. The ill effects of non-adherence are compounded by lifestyle issues such as weight gain and diminishing cardiorespiratory reserves, which generally occur during and after completion of cancer therapy [26, 32–37]. This pattern is not just found in women with breast cancer: there is a growing recognition that this pattern is also common in both genders and other cancer populations [38–48]. Consequently, compared to individuals without a cancer history, individuals with cancer have disproportionately higher burdens of CVD [46, 49–52].

These shortfalls in coordination of care, medication adherence, lifestyle changes, and focus on optimally controlling CVD risk factors highlight an urgent need for health care redesign. The cancer survivors’ PCP must become an integrated member of the cancer care team. The status quo of simply telling patients to follow-up with their PCP is insufficient. PCP follow-up is also inadequately addressed in most survivorship care plans. Therefore, we propose to implement an onco-primary care model and engage the PCP as an active member of the cancer team. The overarching goal of the Onco-primary care networking to support TEAM-based care (the ONE TEAM study), is to optimize the management and outcomes of individuals with cancer, both during and after treatment, and to develop a ‘low-touch’ multi-level intervention that can be generalized, adapted, and scaled in other health care systems with or without a survivorship clinic.

## Methods/Design

**Fig. 1:**
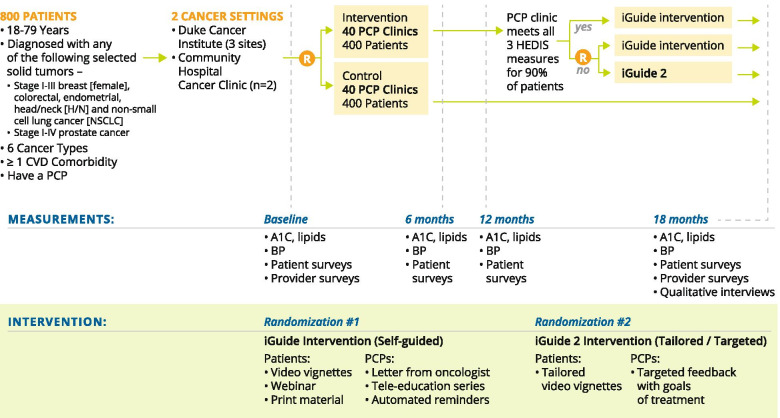
Design of the ONE TEAM STUDY

The ONE TEAM study is an 18-month clustered randomized controlled trial with a sequential multiple assignment randomized trial (SMART) design (Fig. 1) (clinicaltrials.gov identifier NCT04258813). We will prospectively enroll 800 patients with one of six newly diagnosed solid tumors (stage I-III breast, colorectal, endometrial, head/neck, and non-small cell lung cancer; stage I-IV prostate cancer) over a 3-year period, comparing a remotely delivered, low touch, patient- (*n*=400) and PCP- (*n* = 80) directed intervention with an education-only control.

To engage the PCP early in the process, we will enroll patients from across North Carolina at the time of one of their first visits with a cancer specialist (e.g., surgeon, radiation or medical oncologist) from two cancer settings (Fig. 1). Most participants will transition from active therapy to follow-up care during the 18-month study period, with the vast majority transitioning within 6-9 months.

The overall objective of ONE TEAM is to determine an optimal intervention that will improve patient outcomes according to the following measures: (1) Healthcare Effectiveness Data and Information Set (HEDIS) quality measures of management of three CVD comorbidities using laboratory testing (glycated hemoglobin [A1c], lipid profile) and blood pressure measurements; (2) medication adherence assessed via pharmacy refill data using Proportion of Days Covered (PDC); and (3) patient-provider communication (Patient-Centered Communication in Cancer Care, PCC-Ca-36 ) [53].

### Participants and randomization

Participants will be recruited from one of five cancer treatment sites at one of their first cancer specialist visits. These sites include three Duke Cancer Institute sites (Duke Medical Center, Duke Raleigh, Duke Regional) and two community oncology practices (Scotland Cancer Center in Lumberton, NC and Gibson Cancer Center in Laurinburg, NC). For the purposes of enrollment, a cancer specialist visit is defined as a visit with the surgeon, radiation oncologist, or medical oncologist. This will allow the research team to engage the participant’s PCP early in the process. We will identify potentially eligible participants in the electronic health record (EHR). If pathologic staging is not available at the time a patient is identified, the study team will hold off on recruitment until staging has been completed. At the time of the first cancer specialist visit, our research staff will introduce the study to the patient with a brief brochure. For interested individuals, our research staff will confirm eligibility and obtain informed consent. We will also collect reasons for non-participation. Following completion of study consent, the research team will collect the study measurements. We anticipate the survey will take most patients approximately 25 minutes to complete. Participants will be given a debit card, which will be loaded with $25 after completing each assessment at baseline, 6-, 12-, and 18-months. Participants will be given up to 2 weeks to complete the survey in person, online via REDCap, or over the phone with a research staff member depending on their preference. Those who are not able to complete the baseline survey within 2 weeks will be withdrawn from the study.

We will enroll a total of 800 individuals who meet the following inclusion criteria:

- ○ Diagnosed with incident Stage I-III breast [female], colorectal, endometrial, and head/neck, Stage I-III non-small cell lung cancer [NSCLC], or stage I-IV prostate cancer
- ○ Treated with curative intent
- ○ 18-79 years old at the time of cancer diagnosis
- ○ Has at least one of three CVD risk factors / comorbidities (hypertension, diabetes, or hypercholesterolemia) – based upon whether the patient is currently on a medication for the comorbidity at time of recruitment
- ○ Had a visit with their PCP in the previous 12 months and has CVD comorbidities managed by the PCP

Individuals will be ineligible if they have had a myocardial infarction in the previous 24 months, have a diagnosis of heart failure with an ejection fraction <30% or of stage IV-V chronic kidney disease (eGFR <30). Participants who progress to metastatic disease during the course of the 18-month study period will be allowed to continue to participate unless they voluntarily withdraw from the study.

We will use a SMART design with two randomizations [54–57]. The unit of analysis will be the patient. The unit of randomization will be the PCP clinic. We selected this approach to avoid contamination between PCPs within a clinic. We will stratify each randomization by category of PCP (Duke PCP, non-Duke PCP). We selected this stratification factor to maintain a balance between the two arms with respect to PCP setting. The first randomization, will occur at enrollment, and participants will be cluster randomized to the self-guided multi-level iGuide intervention or control arm.

The second randomization will occur at 12-months [55, 57], and we will use an embedded dynamic treatment regimen [54, 55] (also referred to as an adaptive intervention ) [58, 59]. PCP clinics in the intervention arm will be considered non-responders if 90% or more of their participating patients do not meet the modified HEDIS quality metrics at the 6-month measurement. These clinics will be randomized to a more intensified and tailored intervention (iGuide 2) or continue on (iGuide 1). We are using the 6-month data to determine the threshold for second randomization eligibility because of the potential timing of patient enrollment. This approach allows additional time for patients to enter the trial and contribute additional data to a given cluster. Assessments will be conducted at study enrollment, 6-months, 12-months, and 18-months. Because of variability in appointment scheduling, we will allow a window of one month for assessment. iGuide 1 consists of two patient-level and four PCP-level components (Table 1).

**Table 1:** Intervention components

	iGuide intervention (Self-guided)	iGuide2 (Tailored/Targeted)
**Patient-level components**	(1) the patient-level brief video vignettes with a written summary describing: (a) importance of heart health, (b) how your primary care provider can help you during and after cancer treatment; (c) heart health: taking your medicines properly; (d) taking control of blood pressure; (e) eating well to maintain your health; (f) keeping your heart healthy by staying active; and (g) life after cancer therapy and managing more than one health problem. (2) patient-facing webinars hosted by a cancer survivor, medical oncologist, primary care provider, and other relevant providers.	Tailored video vignettes with pre-video tip sheets designed to inform patient about: (a) value-based goal setting, (b) readiness and self-efficacy for chronic disease management, (c) taking medication as prescribed, (d) and preparing for an office visit.
**PCP-level components**	(1) a brief letter from our research team notifying the PCP that their patient has enrolled and a brief description of the study; (2) a brief EHR-templated letter from the cancer care team notifying the PCP of the patient’s cancer diagnosis; and indicating the cancer team and PCP’s roles in in managing the patient’s conditions; (3) a monthly tele-education, case-based series that covers case management recommendations from oncology experts to help expand the PCP’s capacity to manage complex diseases; (4) quarterly automated treatment update messages from the cancer team sent through the EHR reinforcing the importance of CVD risk factor management	(1) cancer specialist-facing dashboard that will be oncologist-specific versions of the study Enrollment report listing each of the HEDIS measures (2) specialist-to-PCP quarterly automated letter offering a case review

### Intervention components

#### iGuide 1: patient-level intervention

Participants randomized to the control group (*n*=400) will receive current guideline-concordant cancer care. We will also provide information for healthy living during and after cancer and for preparing for transition from cancer therapy to follow-up care. Monthly, patient education material on healthy living will be sent to the participants via the patient portal or by mail, based on participant’s preference. Near the completion of therapy, they will be provided the NCI Facing Forward: Life After Cancer booklet [60]. PCPs will receive a brief letter from our research team notifying them that their patient has cancer and has joined the study. They will also be asked to complete baseline and end of study surveys. This approach is not fully equivalent to an attention control as there will be touch points in the iGuide 1 and iGuide 2 interventions that we cannot match for the control group. Also, we will not engage the PCPs in clinics randomized to the control group.

Participants in the multi-level intervention will receive the iGuide 1 interventions. These patient-level components include seven brief video vignettes with a written summary and a live webinar. The scripted 3-minute video vignettes (one per month) will cover the following topics: (1) overview and importance of managing CVD risk factors; (2) role of the PCP during and after cancer care; (3) importance of medication adherence to prevent CVD events; (4) blood pressure control; (5) healthy diet for cancer and prevention of heart disease; (6) physical activity for cancer survivors and prevention of heart disease; and (7) transitioning off of therapy and managing other comorbidities. Each of the vignettes will be accompanied by a printed, one-page, bulleted summary, written at the 7^th^ grade reading level. In accordance with a patient’s preference, all materials will be available via the patient portal (MyChart), online streaming, and on a USB drive.

We will also conduct one patient-facing webinar for each recruitment group (50 minutes). Each live webinar will discuss the importance of managing CVD risk factors during and after cancer therapy and will include a moderator, a cancer specialist, a PCP, and a cancer survivor. The panelists will provide their perspective on the importance of managing non-cancer comorbidities during therapy and the role of the PCP during and after cancer therapy. Following these short perspectives, there will be a question and answer session. Survivors in the iGuide 1 Intervention arm will be invited to one webinar. If a participant cannot attend the webinar, a recorded copy of the webinar will be sent on a USB drive, and the participant will be invited to the next webinar (with the next group). All webinars will be recorded and can be watched again with video plus audio or audio only.

#### iGuide 1: PCP-Level Intervention

There are four components in iGuide 1 at the PCP-level: (1) a brief letter from our research team notifying the PCP that their patient has cancer and has enrolled in our study; (2) a brief letter from the cancer care team asking the PCP to actively manage CVD comorbidities during and after cancer treatment; (3) invitations to a monthly tele-education, case-based series with free Continuing Medical Education (CME) credits; (4) and quarterly automated treatment update letters from the cancer care team reinforcing the importance of CVD risk factor management. These letters will be delivered through Epic as an InBasket message for Duke PCPs and by autofax for outside PCPs. We have partnered with Duke Office of Clinical Research (DOCR) to develop an efficient workflow approach requiring a minimum of steps and avoiding EHR fatigue. The letters to the PCP from the research team and cancer care team will be automatically sent by our EHR at the closing of the participant encounters (at baseline, months 6, 12, 18) or letter encounters (at months 3, 9, 15).

The PCPs with patients in the intervention group will be invited to a monthly, 45-minute case-based, tele-education series. The format for the series has been adapted from Project ECHO (Extension for Community Healthcare Outcomes) a validated method that expands PCP capacity to manage complex diseases by sharing knowledge, disseminating best practices, and building a community of practice [61–65]. In designing the format, we adopted the implementation tools and best practices developed by Serhal et al. [66] A ‘Hub’ and ‘Spoke’ model will be used wherein the research team and cancer specialists at Duke Cancer Institute and our two community oncology practices will serve as content experts (the hub), and PCPs in the intervention arm will be the spokes. In this approach, a short didactic lecture is delivered by a member of the hub team and recommendations for case management are offered by the community in response to anonymized clinical cases presented by the spoke sites. PCPs will receive Continuing Medical Education credit for each session attended.

### iGuide 2

At the 12-month time point, we will assess the three HEDIS measures for CVD risk factors for the survivors in the intervention arm, using fasting laboratory values (A1c, lipid profile) and blood pressure measurements performed by our research staff, as well as other key measures shown in Table 2. We will determine which PCP clinics do not have at least 90% of their participants meeting all three HEDIS quality measures based on all available 6-month assessments. We set the 90% bar recognizing that some clinics may have only a few participants. PCP clinics (and their patients) not meeting this threshold will be considered ‘non-responders’ and randomized to either the iGuide 2 intervention or to continue on the iGuide 1 intervention.

**Table 2:** Key measures

Measures	Definition / Criteria	Data source
Primary outcomes		
HEDIS measures [67–69]	BP <140/90 mm Hg A1c <8.0% On statin if diabetic or ASCVD risk > 10%	BP by research staff, fasting labs, EHR
Proportion of days covered [70, 71]	Ratio of the number of days the patient is covered by a mediation during a refill period	EHR
PCC-Ca-36 [53]	Patient-centered communication: exchanging information, making decisions, fostering healing relationships, enabling patient self-management, managing uncertainty, and responding to emotions	Patient self-report survey
Secondary outcomes		
Voils’ medication adherence self-report measure [72, 73] (modified)	Measure of adherence & reasons for non-adherence of medication for key CVDs	Patient self-report survey
Haggerty et al [74] (modified) Vimalananda et al. [75] (modified)	Multiple perspectives of care coordination	Patient- and provider- survey
FACIT-COST [76]	Cancer care-related financial toxicity	Patient self-report survey
Objective self-report measure [77]	Amount of out-of-pocket expense on care by spending type (e.g., medication, copayments, etc.)	Patient survey
Patient activation measure (PAM) [78]	Patient engagement	Patient self-report survey

#### iGuide 2 Patient-level Intervention

For the iGuide 2 patient-level intervention (Table 1), we will use a tailored approach. Patients will receive four monthly, 5-minute video vignettes that incorporate a pre-video tip sheet. We will send the tip sheets and video vignettes in the method preferred by the patient (as noted above), using the patient portal, online streaming, or a USB drive with printed versions of the tip sheets. In developing the video vignettes, we will use motivational interviewing and goal setting techniques [79, 80]. Based upon responses, the patient will select which video vignette to watch (i.e., who is most like me). Patients will be able to watch any of the other videos they choose. The topics for the four video vignettes will be: (1) value-based goal setting; (2) tips based on readiness; (3) tips to take your medicine as prescribed; and (4) preparing for an office visit. At the end of each video, patients will be asked to create a list of one or two items to discuss with their PCP.

#### iGuide 2 PCP-level intervention

We will implement a cancer specialist-facing EHR dashboard that includes the specialists’ patients who are enrolled in the study and are in the intervention arm. The HEDIS quality measures for our three CVD risk factors will be used. The dashboard will be populated with data available in the EHR for the specific patient. Quarterly, starting with the second randomization, an automated asynchronous specialist-to-PCP letter will be sent to iGuide 2 PCPs  offering a case review.

#### Dissemination to participants

At the end of the study both patients and PCPs will be mailed a newsletter with a summary of the study findings. In addition, patients in the control group will be sent a copy of the printed materials along with a USB drive with the video vignettes and a recorded webinar.

### Statistical considerations

The aims of this study will be analyzed as intent to treat. To account for the study design, we will use longitudinal mixed models. Each model will specify fixed effects for both intervention (i.e. control, iGuide 1, or iGuide 2) and time point (i.e. enrollment, 6-months, 12 months, and 18 months). An interaction between the fixed effects of time point and treatment will be included. Time point and cluster (PCP location) will be included as random effects.

For each of the three primary aims, a separate model will be built. The HEDIS measurement will be included as a binary variable (criteria met vs. not met). For this model, a binomial distribution with a logit link will be specified. Medication adherence, defined as percent days covered (PDC), will be modeled as a continuous outcome with a Gaussian distribution and identity link. Similarly, the patient-centered communication survey (PCC-CA-36) will be modeled as a continuous outcome. From the PCC-CA-36 data, an overall score will be computed as the average of all questions consistent with recommendations from the developers [53]. Secondary aims include self-reports of medication adherence, cancer care-related financial toxicity, out-of-pocket expenses by category of spending (e.g. medication), engagement, and care coordination. Because of the subjective nature of these endpoints, we will categorize each of these variables into quartiles and then analyze them in multivariable analysis using logit regression models with the top quartile category as the comparator and the remaining three quartiles as the reference group.

After each model is built, the primary hypotheses will be tested by constructing a contrast of effects over the period from enrollment to 18 months comparing iGuide 1 and iGuide 2 versus control.

### Sample size calculations

This is a cluster-randomized trial design with a binary outcome, assuming 32 PCP clinics per arm with 10 subjects per clinic, alpha=0.05, and intra-cluster correlation (ICC) ranging from 0.05 to 0.10, assuming at 18 months that the control arm patients will have 50% compliance on the 3 HEDIS measures and the intervention arm will have 65% compliance. With this combination of factors, the power ranges from 0.80 to 0.89, depending upon ICC level. If we assume approximately 20% drop-out due to death or loss to follow-up, we require 40 PCP clinics per arm with an average of 10 subjects per clinic.

## Discussion

Informed by implementation science, the ONE TEAM study is intended to meaningfully change the longitudinal care of cancer survivors by coordinating care with primary care physicians and empowering patients. Currently, cancer survivors are often lost-to-primary care, and as such, they are not being monitored for non-cancer related comorbidities and potential risk factors and behaviors. Compounding the problem, cancer treatments often exacerbate underlying cardiovascular disease and have other transient effects, as do underlying issues associated with developing cancer, such as obesity and smoking. Further, cancer treatments can result in new negative behaviors, such as poor diet and lack of exercise, which can also affect cardiovascular comorbidities, diabetes, and other diseases. The result of all these factors is that cancer survivors die from co-morbidities earlier and at a greater frequency than their counterparts who have not had cancer [2, 3].

To counter this, ONE TEAM was designed to be a low touch delivered intervention, designed to help both the patient and the PCP feel more comfortable with post-cancer care and to help increase adherence to non-cancer medications. One limitation of this study is that our cohort is limited to those who have a PCP, and more work will need to be done to engage those without a PCP. However, because of its relatively low human resource use, if effective, ONE TEAM could be scaled-out in other settings, be used in low resource settings in a variety of age groups across diverse populations.

## Conclusions

As the population of cancer survivors grows, ONE TEAM will contribute to closing the CVD outcomes gap among cancer survivors by optimizing and integrating cancer care and primary care teams. ONE TEAM is designed so that it will be possible for others to emulate and implement at scale.

## Data Availability

N/A: Data and materials have not been developed yet. To ensure participant privacy, data will not be openly shared. All Principal Investigators will be given access to the cleaned data sets.

## Abbreviations

CVD: Cardiovascular disease
DCI: Duke Cancer Institute
EHR: Electronic health record
NSCLC: Non-small cell lung cancer
ONE TEAM: Onco-primary care networking to support TEAM-based care
PCP: Primary care provider
